# Functional Properties of Natural Products and Human Health

**DOI:** 10.3390/nu15132961

**Published:** 2023-06-29

**Authors:** Paola Bontempo, Luigi De Masi, Daniela Rigano

**Affiliations:** 1 Department of Precision Medicine, University of Campania Luigi Vanvitelli, Via L. De Crecchio 7, 80138 Naples, Italy; paola.bontempo@unicampania.it; 2 National Research Council (CNR), Institute of Biosciences and BioResources (IBBR), Via Università 133, 80055 Portici, Italy; luigi.demasi@ibbr.cnr.it; 3 Department of Pharmacy, School of Medicine and Surgery, University of Naples Federico II, Via Domenico Montesano 49, 80131 Naples, Italy

Natural products (NPs), broadly defined as chemicals produced by living organisms including microbes, marine organisms, animals, fungi and plants, are widely used as therapeutic agents for treating diseases and maintaining health and “wellness”. NPs are regarded as an important repository for the development of potential novel drugs, as they may serve as either drug candidates or lead compounds for drug design. NPs have evolved over millions of years and acquired a unique chemical diversity, occupying a much greater chemical space than those created through synthetic chemistry methods, which consequently results in the diversity of their bioactivities and drug-like properties [1]. Plant-derived therapeutics, which may reach the pharmaceutic market as pure compounds or as complex mixtures containing thousands of different molecules, have several advantages, including wide availability, diverse pharmacological actions and a generally good profile of safety and tolerability. As novel therapeutic targets have emerged in recent times, the development of state-of-the-art technologies can be applied to drug discovery from natural sources. Therefore, there have been numerous reports from clinical studies testifying to the efficacy of medicinal plants and phytochemicals in ameliorating several human diseases such as cancers and inflammatory, cardiovascular, neurodegenerative, metabolic and infectious diseases. A plethora of basic in vitro and in vivo studies have also unraveled molecular mechanisms underlying NPs’ health benefits [1].

In this Special Issue, eighteen original research articles are collected, of which one brief report and one review, on new perspectives on the chemical and functional properties of NPs, including the molecular mechanisms of the therapeutic effects of NPs in different models of human diseases.

Research on the plant resources allowed the isolation of a myriad of NPs with anti-inflammatory effects in vitro. Among these, Ma et al. [2] recently studied the bioactivity of the phenolic compound cryptochlorogenic acid (CCGA), a structural isomer of chlorogenic acid obtained from *Ageratina adenophora*, on the inflammatory response induced by lipopolysaccahride (LPS) in macrophages (RAW264.7), also clarifying the molecular mechanisms by which CCGA has shown in vitro anti-phlogosis potential. Moreover, *A. adenophora* is a toxic and invasive species in China (native to Central America), but nowadays it is considered a new bioresource for the discovery and production of specialized metabolites, such as CCGA.

Liu et al. [3] highlighted the beneficial effects of dietary procyanidins against pyroptosis, a type of inflammation-associated programmed cell death with a crucial role in liver diseases. These authors demonstrated that Procyanidin B2 (PCB2), a natural flavonoid known for its antioxidant and anti-inflammatory properties, attenuates pyroptosis induced in hepatocytes by nicotine, a natural alkaloid of tobacco that plays a fundamental role in the development of many diseases. The authors elucidated the role of nicotine in the pyroptotic death of hepatocytes and the mechanism by which PCB2 exerts its antipyroptotic and hepatoprotective effect through the activation of the peroxisome proliferator-activated receptor γ (PPARγ).

Phenolic compounds have been strongly linked with beneficial effects in many in vitro studies of human and animal models, as also confirmed by the other research described in this Special Issue.

Park et al. [4] demonstrated the ability of phenolic compounds to prevent the negative effects on the skin of blue light, also known as high-energy visible (HEV) light. Particularly, they studied the protective effects of yellow chaste weed (YCW) (*Helichrysum arenarium*) extract and its main components, apigenin and galangin, on HEV light-irradiated HaCaT keratinocytes from human skin. They found that YCW extract attenuated (i) the proliferation arrest of HaCaT cells and (ii) levels of reactive oxygen species (ROS). Furthermore, YCW extract, apigenin and galangin were able to modulate TRPV1/clusterin/FoxO3a and MAPK signaling. Therefore, they could be used as potential agents for counteracting the effects of HEV light in the protection of human skin.

Another phenolic compound, pinostrobin, a flavanone found in honey and propolis, was shown to be a potential melanogenic agent to be used in the treatment and prevention of vitiligo and other depigmentation disorders, for which, to date, the only therapeutic options are mostly based on immunosuppressive agents and UV light treatment [5]. This molecule has a stimulating effect on the activity of tyrosinase and on the production of melanin. Molecular docking analysis revealed that it possesses an appropriate molecular structure for effective interactions with major melanogenesis proteins.

Two papers in this Special Issue deal with the neuroprotective effect exerted by NPs. Pattarachotanant et al. [6] studied the effect of *Aquilaria crassna* (AC), a plant used in many traditional medicines, on the neurotoxicity and aging induced by high glucose. Through in vitro and in vivo studies, these authors demonstrated that AC leaf hexane extract (ACH) induces interesting neuroprotective activities in SH-SY5Y human neuroblastoma cells, including induction of neurite outgrowth and cell cycle regulation. In vivo studies on the nematode *Caenorhabditis elegans* have highlighted the beneficial effects of ACH on antioxidant activity, longevity and life span. The authors identified sitosterol and stigmasterol as the bioactive phytochemicals in ACH. Tan et al. [7] showed that the extract of *Pandanus amaryllifolius*, widely distributed in tropical and subtropical environments, has anti-amyloidogenic activity and promotes neuroprotective effects in amyloid-β-induced SH-SY5Y cells, through the restoration of cell viability, reduction in ROS and mitochondrial dysfunction. Furthermore, the unprecedented isolation of nicotinamide as a potential bioactive constituent, provides new insights into the promising potential of *P. amaryllifolius* extracts against Alzheimer’s disease and suggests further exploration of other potential bioactive constituents.

Nowadays, when multidrug-resistant bacterial strains emerge and designing new antibiotics is a long process, rapidly developing a new possible line of infection treatment is essential. Clear evidence of the possible antimicrobial properties of NPs was shown by Nowicki et al., who evaluated the activity of dietary isothiocyanates, particularly sulforaphane and phenethyl isothiocyanate, against a *Shigella dysenteriae* infection in the *Galleria mellonella* larvae model [8]. These authors elucidated the mechanism behind the observed antibacterial effect, which involves induction of global stress response, stringent response, and decrease in Shiga toxin gene expression. The results here described constitute an important step in the perspective of using plant-derived NPs to treat bacterial infections.

Other research showed the potential of NPs to stimulate immune functions and to treat immune-related pathologies. Particularly, Ishiguro et al. [9] demonstrated that the water extract from the cell walls of *Chlorella sorokiniana*, a unicellular green alga used as a dietary supplement worldwide, could be useful to stimulate anti-microbial or anti-tumor immunity. The extract stimulated the growth of bone marrow cells and splenocytes, and increased cytokine mRNA associated with T-cell activation.

These, and other papers, clearly show that plant species represent an apparently inexhaustible resource of bioactive compounds. In spite of this, they can be susceptible of more or less wide variability in phytochemicals and, consequently, in the related bioactivities. The variability of the NP composition in the starting plant material due to environmental factors is a major challenge for the development of botanical drugs. An important line of research in this regard concerns the study of plant metabolome changes in response to variations in environmental conditions. The seasonality of plant harvesting is among the most important factors influencing the bioactive content, and so it deserves to be studied more in depth. In this regard, the in vitro study by Pucci et al. [10] showed that the extracts of common fig (*Ficus carica*) leaves collected in different seasons differently modulate lipid metabolism and adipogenesis in the cellular model of 3T3-L1 adipocytes. Importantly, dysfunctions related to adipogenesis and metabolism are very common in the population, so it would be advisable to utilize the fig autumnal extract, characterized by a higher coumarin content, to correctly downregulate the transcriptional pathway of adipogenesis, as shown in 3T3-L1 adipocytes.

The papers cited above describe noteworthy results, but they face limitations that, as most of the studies on NPs biological activities, are typically performed only through in vitro assessment. If the in vitro studies represent an essential prerequisite to advance the research on the biological effects of NPs, a complete understanding of the in vivo mechanism(s) is necessary and requires advanced studies.

Only a few natural products have been appropriately studied in models in vivo; among them, there is α-bisabolol, a monocyclic sesquiterpene derived from essential oils of many plants, whose pharmacological properties, including anticancer, antinociceptive, neuroprotective, cardioprotective and antimicrobial, were demonstrated based on both in vitro and in vivo studies [11]. Other interesting in vivo studies, described in this Special Issue, regard the potential role of NPs in the treatment of obesity and related diseases, a widespread public health issue all over the world. Increasing amounts of knowledge demonstrate that NPs can modulate metabolic syndrome and its risk factors with limited side effects. Investigations on the in vivo model of obese mice showed that sap of *Dendropanax trifidus*, a medicinal herb native to East Asia, can suppress body weight increase and stimulate energy metabolism in C2C12 muscle cells [12]. Estrogen receptor alpha is present in skeletal muscle cells, so sap containing estrogen as a major component could contribute to controlling body weight by modulation of cellular metabolism via this receptor. This study showed that cellular components could mediate glycolytic and metabolic changes within the cell, thus contributing to weight loss in mice treated with *D. trifidus* sap. Of particular importance in this respect is the gut, an endocrine organ producing the hormone peptide incretins, known for the positive modulatory effects on insulin production, glycemia, lipidemia, gastrointestinal motility, appetite and inflammation. Among incretins, glucose-dependent insulinotropic polypeptide (GIP) could represent a new potential target for treating diabetes, obesity and cardiovascular diseases. The seeds of tea (*Camellia sinensis*) contain saponins, bioactive compounds that have been found to regulate gastrointestinal motility and body weight. Their effects on GIP production, the underlying mechanism and signaling network were investigated in a study in vitro and in vivo [13]. This research showed how tea seed saponins are able to significantly increase GIP levels, both in the small intestine of normal mice and in the mouse intestinal endocrine STC-1 cells, through important mediators of intestinal metabolism.

Stress-induced alterations in the internal homeostasis maintained by the autonomic nervous system can induce hypertension and arteriosclerosis leading to a deterioration of cardiovascular health. Therefore, using the biological effects of NPs, research studies how to reduce the blood levels of cholesterol and prevent the progression of cardiovascular diseases. The findings of an innovative line of frontier research [14], reported in this Special Issue, showed the effects of essential oil (EO) from common basil (*Ocimum basilicum*) and its major compound linalool through olfactory stimulation of the nervous system on stress-induced dyslipidemia in the in vivo model of chronically stressed rats. The interesting results of this study revealed that the simple inhalation of basil EO (i) decreases body weight gain and blood levels of cholesterol, triglycerides, LDL, while (ii) increases HDL and ameliorates atherogenic index and cardiac risk factors. All these beneficial effects should be further explored as soon as possible in clinical trials. Another critical point in the studies about NPs’ therapeutic properties is infact that very few have moved to clinical trials. In the study by Oue et al. [15], the effect of acute dietary NO_3_^¯^ supplementation with beetroot juice on the venous vascular response and circulatory responses to exercise was analyzed on sixteen healthy young adults in a randomized crossover design. The results showed that the treatment leads to an increase in plasma of NO_3_^¯^ concentration but does not change the venoconstriction in non-exercising limbs or the mean arterial pressure response to exercise.

In recent years, the popularity of fermented foods has increased in the Western world, largely due to the emerging evidence that secondary metabolites derived from fermentation-based products provide health benefits. To date, research indicated that fermented foods and beverage contains numerous bioactive substances, originating from the material used, but also resulting from the enzymatic transformations of organic compounds carried out by microorganisms or other techniques [16]. Two examples of research in this Special Issue showed that, as a result of the biochemical transformations of organic compounds by microorganisms, it is possible to obtain foods with not only extended shelf life and microbiological stability, but also a higher nutritional value. Particularly, Jakubczyk et al. [17] have studied the effects of fermentation time and type of Chinese tea (black, green, white and red) on the content of micronutrients in Kombucha, a low-alcohol beverage made by fermenting a sugared tea infusion with symbiotic cultures of bacteria and yeasts (SCOBY), commonly called “tea fungus”. The authors demonstrated that the type of tea, as well as the days of fermentation, have a significant effect on the concentrations of selected minerals, and so Kombucha can represent a good supplement of micronutrients in the human diet. Song et al. [18] showed the in vivo bioactivities of fermented Chinese green tea and spores of the fungus *Eurotium cristatum*. Particularly, the methanol extract of the fermented tea and spores of *E. cristatum* both showed potent lipid-lowering activity in the blood of a high-fat-diet-induced hyperlipidemia model in golden hamsters and significantly reduced the accumulation of fat granules in the liver. Interestingly, the extract and *E. cristatum* spores share similar secondary metabolites, such as the new indole-containing diketopiperazine alkaloid, variecolorin P and four of its analogs.

Another important aspect to consider for a better exploitation of NPs therapeutic effects is that several factors could hamper the bioavailability and absorption of NPs in host cell systems and target sites, such as food matrix, environmental factors, molecule size and the association with gastrointestinal (GI) material. Nowadays, the remarkable progress in the use of encapsulation technologies is providing immense opportunities to provide stable and health-promoting compounds for different fields. In the study by Chen et al. [19], a chitosan-thioglycolic acid (CT) was developed as a physical barrier in the gastrointestinal tracts to inhibit nutrient uptake, exhibiting a superior mucoadhesive property compared to chitosan both in vitro and in vivo. Results show that CT could be exploited as a potential mucosal gel for obesity treatment, as hesperidin encapsulated in CT (CTH) not only effectively reduced mice body weight by 40.91% compared with the high-fat diet group, but also reduced the accumulation of body fat. Moreover, Enache et al. [20] designed two co-microencapsulated delivery systems, containing anthocyanins from cornelian cherry (*Cornus mas*) and the probiotic species *Lacticaseibacillus casei* 431^®^, into biopolymeric whey protein isolated-based materials, including casein and inulin. The selected microencapsulation matrices have unique properties, both from technological and nutritional points of view, and could be exploited to preserve the chemical stability during the shelf life of nutraceuticals or food products, and for a controlled release of bioactive compounds in a GI environment.

In conclusion, NPs represent a promising chemical pool for the discovery of novel drugs. Nevertheless, it is important to underline that issues such as NPs accessibility, sustainable supply and bioavailability require further study for NP-based drugs to continue making major contributions to human health and longevity. Furthermore, phytochemicals are generally classified as safe compounds as they are present in plants and produced naturally; however, the assessment of toxicity risk requires full consideration to eliminate possible toxic reactions and ensure not exceeding the recommended dose.
